# Editorial: Women in obstetric and pediatric pharmacology: 2023

**DOI:** 10.3389/fphar.2024.1431937

**Published:** 2024-05-24

**Authors:** Ariela Hoxha, Nicoletta Mainini, Silvia Visentin

**Affiliations:** ^1^ Internal Medicine Unit, Thrombotic and Hemorrhagic Center, Department of Medicine, University Hospital of Padua, Padua, Italy; ^2^ Neonatal Intensive Care Unit, Department of Women’s and Children’s Health, University Hospital of Padua, Padua, Italy; ^3^ Department of Woman’s and Child’s Health, University of Padua, Padua, Italy

**Keywords:** gender, female, research, equity, pharmacology

The global disparity in gender representation within the field of research, particularly in science, is a concerning issue highlighted by recent UNESCO data, indicating that less than 30% of researchers worldwide are women. Despite having leadership aspirations like their male counterparts, women in science face significant barriers that impede their advancement. Biases and gender stereotypes exacerbate these challenges, leading many talented female researchers to leave the scientific community, especially those facing additional discrimination due to factors such as race, ethnicity, disability, or sexual orientation. This Research Topic aims to showcase the diverse contributions of female researchers across all aspects of pharmacological research to address these inequities and promote the work of women scientists in obstetric and pediatric pharmacology.

Preeclampsia (PE) is a serious condition affecting around 10% of pregnancies globally, characterized by maternal systemic vascular and renal dysfunction, posing risks to both maternal and fetal health. While the primary risk factors for PE are known, the mechanisms linking placental ischemia to the development of maternal complications are not fully understood.

Luizon et al. researched the molecular mechanisms underlying endothelial dysfunction in patients with PE. They highlighted the role of visfatin/eNAMPT, an adipocytokine that inhibits nitric oxide (NO) formation, leading to increased sFlt-1 levels and oxidative stress, common features observed in PE. Additionally, matrix metalloproteinase-2 and tissue inhibitors of metalloproteinase-3, crucial for embryogenesis, placental development, and cardiovascular and renal function, were discussed. The balance between these enzymes in regulating the extracellular matrix is vital, suggesting that their epigenetic regulation may influence endothelial dysfunction and contribute to cardiovascular and renal complications in PE.

Furthermore, Gajic et al. discuss the potential molecular targets of hydroxychloroquine (HCQ) and its effects on biological processes related to preeclampsia (PE). HCQ, an analog of chloroquine, is commonly used to treat systemic lupus erythematosus and rheumatoid arthritis. Its primary mechanism of action involves alkalizing lysosomes. In the context of PE, HCQ is suggested to inhibit Toll-like receptor (TLR) activation through different mechanisms, reducing inflammation, improving endothelial function, and affecting placentation in preeclamptic patients. Furthermore, HCQ may modulate the adaptive immune system’s response in PE by influencing cytokine production, shifting T cell balance towards Treg and Th2 responses, and hindering MHC II molecule expression through its effects on endosome pH. These mechanisms suggest HCQ as a promising candidate for PE treatment due to its multifaceted impact on inflammatory pathways and immune responses involved in the pathogenesis of the condition.

Bouhuys et al. focused on ustekinumab use in pediatric Crohn’s disease cases refractory to anti-tumor necrosis factor (anti-TNF-α) therapy. They evaluated ustekinumab trough levels, C-reactive protein levels, and fecal calprotectin before each drug administration in six adolescent patients. Standard adult ustekinumab dose was successfully achieved in two patients as biochemical remission in one (reduced fecal calprotectin levels below 250 mg/kg) and clinical remission (the resolution of symptoms) in another patient. Four patients did not respond adequately to standard dosing; intravenous re-induction and shortening the dosing interval were employed to increase ustekinumab trough levels. This adjustment resulted in biochemical remission for one patient and clinical remission for another, indicating a possible relationship between drug exposure and response. These findings suggest that escalating the dose before deciding to discontinue the drug due to perceived ineffectiveness may be warranted in some cases. This study demonstrates that ustekinumab holds promise for inducing remission in pediatric patients with Crohn’s disease who are resistant to anti-TNF-α. Prospective studies are needed to assess the long-term efficacy of ustekinumab and the utility of therapeutic drug monitoring approaches to define optimal target trough levels.

The original article by Esposito et al. highlights the critical relationship between preterm birth and the onset of *postpartum* depression, which can have significant implications for the health and wellbeing of mothers, infants, and families. Infants born to mothers experiencing *postpartum* depression are also at elevated risk for adverse developmental outcomes. This population-based study conducted using regional health databases in Lombardy revealed that among mothers with no history of depression for at least a year before conception, there was a 0.9% risk of initiating antidepressant treatment in the first year *postpartum*. This risk increased by 0.3% in cases of moderate to late preterm births and by 0.9% in instances of extremely and very preterm births. Understanding and addressing the complex interplay between preterm birth, *postpartum* depression, and antidepressant use is crucial for ensuring the wellbeing of both mothers and infants. This research underscores the importance of early identification, intervention, and support for women at risk of *postpartum* mental health challenges, particularly those who have experienced preterm birth, to mitigate the potential adverse outcomes associated with these conditions.

Principi et al. review the critical issue of antibiotic overuse contribution to developing bacterial antimicrobial resistance, focusing on pediatric respiratory infections. Current evidence does not conclusively support short-term antibiotic therapy for acute streptococcal pharyngitis. While further research is necessary to define appropriate short-term antibiotic regimens and assess their clinical efficacy, safety, economic implications, and impact on long-term complications such as acute rheumatic fever, the paper highlights the need for a more nuanced approach to antibiotic prescribing in these cases. Uncertainties exist regarding the specific antibiotic choices and the effectiveness of shorter courses, especially in younger children, for acute otitis media. In pediatric cases of community-acquired pneumonia, short-term antibiotic therapy has demonstrated comparable efficacy to longer courses with lower rates of adverse events. Meta-analyses have indicated that short courses are non-inferior to longer regimens, particularly in children under 5-years-old. Short-term therapy has shown benefits in reducing gastrointestinal adverse events and developing bacterial resistance. This review suggests that the evidence supporting short-term antibiotic therapy in pediatric respiratory infections raises the possibility of revising guidelines to recommend this approach for mild to moderate cases.

This Research Topic gathered many studies highlighting the competence of female researchers across all aspects of pharmacological research. These studies emphasize that we are on the road to success in achieving gender equality, diversity, and inclusion in research and publishing is on the road.

